# Modelling group navigation: transitive social structures improve navigational performance

**DOI:** 10.1098/rsif.2015.0213

**Published:** 2015-07-06

**Authors:** Andrea Flack, Dora Biro, Tim Guilford, Robin Freeman

**Affiliations:** 1 Department of Zoology, University of Oxford, South Parks Road, Oxford OX1 3PS, UK; 2 Institute of Zoology, Zoological Society of London, Regents Park, London, UK

**Keywords:** collective navigation, social networks, collective decision-making, self-propelled particles, bird flocks

## Abstract

Collective navigation demands that group members reach consensus on which path to follow, a task that might become more challenging when the group's members have different social connections. Group decision-making mechanisms have been studied successfully in the past using individual-based modelling, although many of these studies have neglected the role of social connections between the group's interacting members. Nevertheless, empirical studies have demonstrated that individual recognition, previous shared experiences and inter-individual familiarity can influence the cohesion and the dynamics of the group as well as the relative spatial positions of specific individuals within it. Here, we use models of collective motion to study the impact of social relationships on group navigation by introducing social network structures into a model of collective motion. Our results show that groups consisting of equally informed individuals achieve the highest level of accuracy when they are hierarchically organized with the minimum number of preferred connections per individual. We also observe that the navigational accuracy of a group will depend strongly on detailed aspects of its social organization. More specifically, group navigation does not only depend on the underlying social relationships, but also on how much weight leading individuals put on following others. Also, we show that groups with certain social structures can compensate better for an increased level of navigational error. The results have broader implications for studies on collective navigation and motion because they show that only by considering a group's social system can we fully elucidate the dynamics and advantages of joint movements.

## 1. Introduction

Travelling collectively can be beneficial for a variety of reasons. Besides, for example, safety in numbers [1,2] and aerodynamic advantages deriving from formation flight [3], flocking may enhance navigational accuracy because collating estimates from multiple individuals can reduce the navigational error of the group [4,5]. This has been supported by several empirical studies on birds [6,7], fish [8] and humans [9]. Theoretical work focusing on interactions between group members has strengthened our understanding of how individual behaviours scale to coherent collective motion (see [10] for a recent review). These studies have demonstrated that synchronized movements of large groups can arise from basic rules determining local inter-individual interactions [11,12]. For example, in simulations, three simple rules (avoid—attract—align) can produce group movements that closely resemble those of real animal collectives [12]. Furthermore, models have been used to examine the effect of moving as a group on navigational accuracy, showing that group membership can be beneficial under certain conditions [10–14]. More specifically, Codling *et al.* [14] showed that individuals' navigational abilities are crucial in determining whether individuals derive benefits from navigating as a group. Yet, whether and how groups with specific social networks can compensate for an increased level of navigational error, and thereby improve group navigation remains to be explored. Until very recently, models examining collective motion and decision-making have neglected the role that underlying social relationships between group members may play in tuning individuals' use of interaction rules in their responses to others. Many social animal groups can be distinguished from aggregations of identical individuals without social networks by the presence of preferred interactions between group members [15]. Preferred connections might arise between familiar conspecifics [16], sexual partners or parents and offspring. For example, some bird species migrate in family units [17] thereby providing additional advantages such as shared vigilance or alliances in conflict situations [18]. Familiarity between individuals can modulate an individual's tendency to follow the movements of a preferred partner [16,19–21]. Incorporating social preferences into a model of collective motion recently showed that social structure can influence group cohesion, the positioning of specific individuals and the movement dynamics within the group [22]. Also, undirected social preferences between group members can improve navigation accuracy and reduce group fragmentation in large, leaderless groups [23–25]. A recent study has found that groups find optimal solutions for different tasks when their members' competence is hierarchically distributed among the group members, i.e. the group consists of a minority of informed individuals [26]. This finding was nearly independent of group size and the structure of the underlying interaction network. Further, using high-resolution GPS tracking, it has been shown that decision-making in flocks of homing pigeons is hierarchically organized, where given pairs of individuals within the group exhibit relatively stable, directed leader–follower relationships [27,28]. This in turn means that some individuals are able to contribute with consistently greater weight to the movement decisions of the flock. Here, we investigate the role of social connections in group navigation by simulating groups that specifically resemble flocks of pigeons in their organization, and thereby draw on previous empirical and theoretical findings [19,28,29]. Pigeons are social birds in all their activities and actively seek out the proximity of conspecifics even during homing flights [30]. Pettit *et al.* [29] studied the interactions within pairs of pigeons to observe distance-dependent attraction, alignment and avoidance responses that support the assumptions of many self-propelled particle models. Although their data also suggest that over short-ranges there might be topological limits to interactions, as data on starling flocks [31] and fish schools [32] suggest, this still has to be demonstrated empirically in larger pigeon flocks. As such, our parameters here are based on known pigeon navigation studies and use a metric interaction model [29,30]. We examine the effect of preferred attachments between certain individuals on the performance of the group in order to test which form of group organization endows the collective with the greatest navigational advantages.

## 2. Material and methods

### 2.1. Group motion model

In order to examine the effect of group organization on navigational accuracy, we extend an existing collective motion model [33] by incorporating internal group structures. We simulate a group of *N* individuals, represented by position vector *c*(*t*) and direction vector *v*(*t*), moving through a two-dimensional environment towards a fixed target location. Individuals interact with other group members within their ‘sensory range’. This sensory range is divided into three interaction zones: avoidance zone (radius *r_R_*), alignment zone (*r_O_*) and attraction zone (*r_A_*). At all times, each individual tries to maintain a minimum distance between itself, *i*, and others, *j*, by turning away from individuals within the avoidance zone:2.1
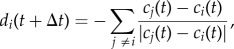
where *d_i_* represents the individual's preferred direction of travel. This behavioural rule has the highest priority. If there are no individuals within the avoidance zone, individuals will be attracted to, and align with their neighbours within the attraction zone and the alignment zone. The preferred direction is calculated as2.2
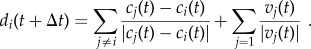
Here *d_i_*(*t* + Δ*t*) is normalized to a unit vector, 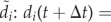 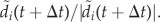 Each individual has information about a target, *g*, simulated as a unit vector from *c_i_* to the target location. The target location is fixed at a random position on a given radius (*r* = 9000 m) around the starting point. The initial positions and velocities were randomly drawn from normal distribution (*μ* = 0; *σ* = 1). While navigating towards this target, individuals must balance their preference to maintain group cohesion with their preferred direction, resulting in their new preferred direction 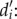
2.3
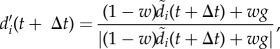
where *w* is a weighting factor between the individual's social attraction and its preferred direction. *w* ranges from 0 to 1 with *w* = 0 implying no navigation towards the preferred direction and *w* = 1 represents only the use of navigational and no social information.

In order to examine the effect of inter-individual (‘social’) relationships within the group on navigational performance, we extend the above model by including a ‘social preference’ factor *h* [23,34]. This affects the relationships between individuals by weighting their interactions:2.4
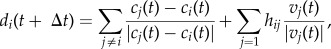
where *h_ij_* represents the social interaction between each pair of individuals. Higher values of *h* will cause attraction and aligning interactions with the given individual, *j*, resulting in it having a greater influence on movements of individual *i* (i.e. leadership).

As in [33], we simulated random influences on an individual's movement. In order to do so, we modified its desired direction 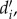 by rotating it by a random angle taken from a circular wrapped Gaussian distribution, centred on 0, with standard deviation *σ* = 0.01 radians, resulting in a new vector 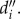 Furthermore, the maximum turning angle of an individual at each time step was *θ* = 0.2 radians. This means that if the angle between *v_i_*(*t*) and 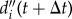 is smaller than 0.2 radians, then they align with their desired vector, 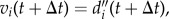 otherwise they turn *θ*Δ*t* towards it. The new position vector of individual *i* is given by *c_i_*(*t* + Δ*t*) = *c_i_*(*t*) + *v_i_*(*t* + Δ*t*)Δ*ts_i_*, where *s_i_* (*s_i_* = 15 ms^−1^) is the speed of individual *i* and Δ*t* is the time step (Δ*t* = 0.1 s). Such a speed value corresponds to a normal flight speed of a homing pigeon. Also, given the speed and the distance to the target, we ensured that the group could reach the goal within the number of iterations (*T* = 600 s). Based on data from real pigeon flocks that has been collected and reported previously [28–30], we confined the parameters to the following: *N* = 10, *r_A_* = 150 m, *r_O_* = 20 m, *r_R_* = 3 m. We used 1000 replicates for each parameter combination and input network.

### 2.2. Social structures of the group

**Figure 1.**
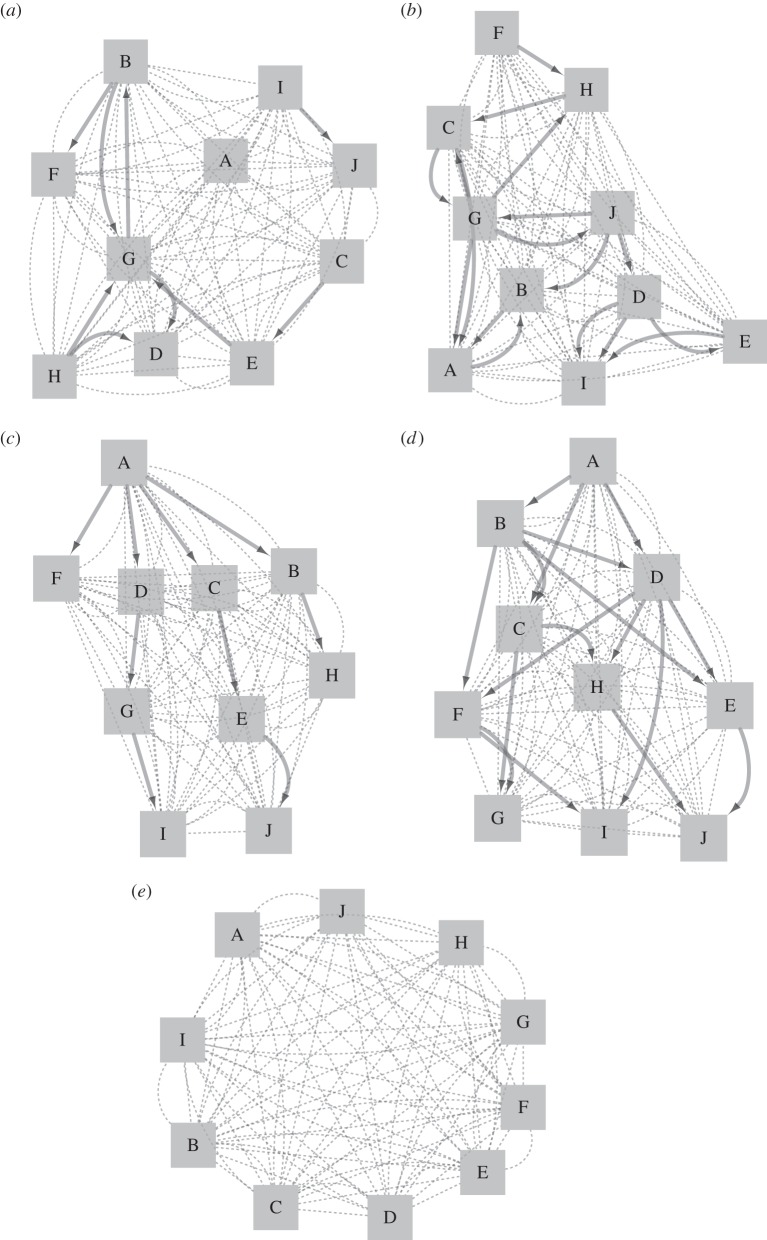
Examples of generated networks used to represent underlying social group structure. The top row shows Erdös–Rényi random, directed models with an average out-degree of (*a*) 0.9 and (*b*) 1.7; the middle rows show directed Barabasi–Albert models with an average out-degree of (*c*) 0.9 and (*d*) 1.7; the bottom row shows a group with no network (i.e. all individuals are connected through weak connections meaning every member is influenced equally by every other member; (*e*) nodes represent individuals. Strong connections are shown as solid edges pointing from the social-leader to the social-follower; weak connections are shown as dotted edges.

Following [23], we restricted the weightings in underlying social preference networks to ‘strong’ and ‘weak’ connections. Individuals react to every conspecific due to the presence of weak connections throughout the group, however strong connections will have a greater impact on the group. Values of *h* are generated to mimic preferred and non-preferred connections between individuals. We set weak connections to *h* = 1 and strong connections to *h* = 100. We developed two types of group structures, based on (i) asymmetrical Erdös–Rényi random, directed models in which strong connections are added randomly [35], henceforth *random network* (figure 1*a*,*b*), and (ii) asymmetrical directed Barabasi–Albert models, which start with a small number of nodes and expand by the addition of new nodes until the final group size is reached. New nodes attach preferentially to already well-connected nodes [36], henceforth *hierarchical network* (figure 1*c*,*d*). These two network types cover many of the possible group structures. Each network is described by its average out-degree, i.e. the average number of strong connections per individual. An average out-degree of 0.9 (in a group of 10 individuals) means that each individual is following one other individual (one strong, directed link per node; figure 1*c*). This value cannot reach 1, because in hierarchical networks the leading individual cannot follow another individual. This means that the maximum number of strong connections within a group of 10 individuals can only be 9. Similarly, a group with an average out-degree of 1.7 has two strong, directed connections per individual (figure 1*d*), yet the top two individuals can follow only one or no other group member, respectively. Within the social structure, highly influential individuals are referred to as ‘social-leaders’ which affect the movements of ‘social-followers’ more strongly than vice versa (figure 1). In groups without network structure (henceforth: *no network*), all individuals are connected through weak connections meaning every member is influenced equally by every other member (figure 1*e*). All simulations and analyses were conducted in Matlab (The Mathworks Inc., Natick, MA, USA)

### 2.3. Data analyses

In order to explore the impact of a group's underlying social structure on navigational performance, we measured navigational accuracy defined as the group's distance to the target location at the end of the simulation (i.e. the further the group from the target after *T* time steps, the lower the navigational accuracy). At the start of each simulation, flocks were initially placed with a mean position at distance such that continuous and direct movement to the goal would result in the highest possible accuracy (zero distance to target). As in [23], we measured for each simulation the probability of fragmentation defined as the proportion of simulations that resulted in more than one group. The radius that defines cohesiveness is *r_A_*_,_ meaning that in a cohesive group all individuals are either directly or through others connected to each other (i.e. within a radius of *r_A_* of each other). Unless otherwise stated, we use Wilcoxon signed-rank tests to assess the significance of differences between groups with different social structures.

## 3. Results

To explore the impact of underlying social structure on navigational accuracy, we measured how closely our groups reached the target. Groups, in which all group members have the same weighting factor (*w* = 0.3) and that do not possess a social structure, missed the target by an average of 509 m (±293 m s.d.). By introducing a social network, this accuracy changed.

**Figure 2.**
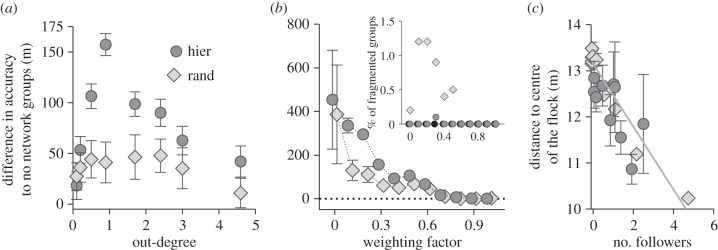
Difference in navigational accuracy between groups with (hierarchical and random) and without underlying network structure (mean ± s.e.m.) as a function of (*a*) average out-degree (*w* = 0.3) and (*b*) weighting factor (out-degree = 0.9). (*c*) Average distance to the centre of the flock (mean ± s.e.m.) as a function of the number of followers for hierarchical and random networks. Navigational accuracy is calculated as the distance of the group's centre of mass to the target at the end of the simulation. Groups with random or hierarchical networks are shown as light grey diamonds and dark grey circles, respectively. Inset shows the percentage of fragmented groups (i.e. those in which not all individuals remained within a distance of *r_A_* from their nearest neighbour) as a function of the weighting factor.

Because networks may vary in the number of strong connections, we first explored how navigational performance changes as a function of its underlying social structure and the degree of connectedness within the group. We increased the number of strong connections from one per group (out-degree = 0.1) to one per individual (0.9) until reaching fully connected groups (4.6 for hierarchical groups). The change in navigational performance of hierarchical groups showed two phases. First, it increased with the number of strong connections until all individuals had one strong connection (0.9). Groups with random social structure did not show this pattern. Whereas hierarchical groups with an out-degree of 0.9 arrived on average 157.3 m (±340 m s.d.) closer to the target than groups with no network structure, random groups improved by only 41.2 ± 635 m (mean ± s.d.). Second, navigational performance of hierarchical groups decreased again after increasing the number of strong connections to more than one per individual (figure 2*a*). Again, this pattern was not observed for random groups. Therefore, to explore the largest possible difference in navigational accuracy between groups with random and directed networks, we focus in all remaining simulations on networks with an average out-degree of 0.9.

### 3.1. Influence of the weighting factor

We examined the effect of different types of internal group structure for different weighting factors, *w. w* ranges from 0 to 1 with *w* = 0 implying no navigation towards the preferred direction and *w* = 1 represents only the use of navigational and no social information. First, each member of the group had the same weighting factor irrespective of its position in the network. We looked at the change in navigational performance, relative to groups with no underlying social structure. Both, the weighting factor (*F*_10,21978_ = 6.9, *p* < 0.001, two-way ANOVA) and the type of network structure (*F*_1,21978_ = 4.1, *p* = 0.04, two-way ANOVA) improved the navigational accuracy of the group (figure 2*b*). Especially at low values of *w*, groups with hierarchical structures improved drastically. Because previous results suggested that leading individuals are located centrally in the group [24], we tested how the number of followers related to the distance to the centre of the flock. Only for hierarchical networks, we found a negative correlation between these two measures (*R*^2^ = 0.5, *p* = 0.027, Spearman; figure 2*c*).

Group fragmentation (i.e. groups in which not all individuals remained within a distance of *r_A_* from their one nearest neighbour) did not occur very frequently in our simulations. Groups with random social structures did occasionally split, but only when the weighting factor was low (figure 2*b*, inset). This is most likely because as *w* increases, navigation dramatically improves. All individuals have the same point target which is why efficient navigation promotes group cohesion. Also, random social structures allow the formation of several unconnected subgroups, which then navigate independently of each other, whereas in hierarchically organized groups every member is connected to every other member.

### 3.2. Relationship between weighting factor and number of social-followers

**Figure 3.**
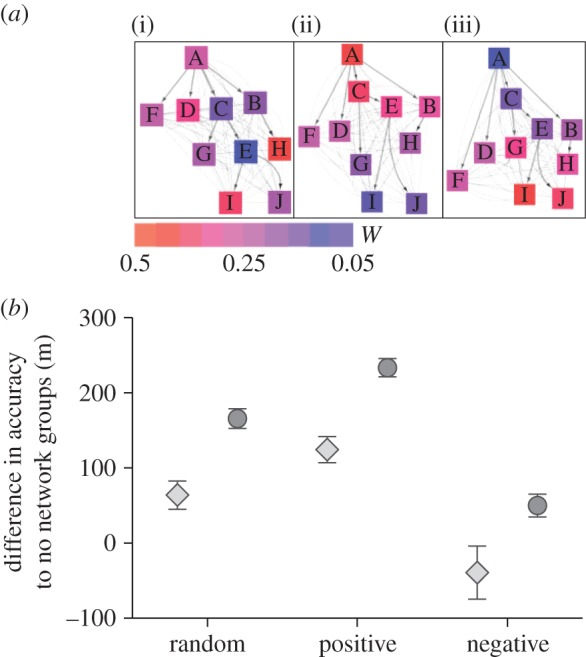
Example of hierarchically organized groups with different relationships between weighting factors and number of followers: (i) randomly assigned, i.e. irrespective of an individual's network position, (ii) positively correlated with individuals' respective number of followers (out-degree), or (iii) negatively correlated with the out-degree. Nodes represent individuals. Colour corresponds to weighting factor (between 0.05 and 0.5). Strong connections are shown as solid edges pointing from the social-leader to the social-follower; weak connections are shown as dotted edges. (*b*) Difference in navigational accuracy between groups with (hierarchical and random) and without underlying network structure (mean ± s.e.m.) as a function of relationship between weighting factor and number of followers for different networks. Groups with random or hierarchical networks are shown as light grey diamonds and dark grey circles, respectively.

We next explored the effect of changing an individual's weighting factor, *w_i_*, depending on its number of social-followers. Because it has been shown that age and experience can influence leadership and flock structuring in pigeons [37,38], we can assume that the weighting factor is not constant among group members. Therefore, we distributed weighting factors in the range of 0.05 to 0.5 among group members (see figure 3*a* for distribution of weighting factors in groups with hierarchical networks; groups with no or random networks not shown). We assigned each individual with a weighting factor, either (i) randomly, irrespective of its network position, (ii) positively correlated with the number of the individual's followers (out-degree) or (iii) negatively correlated with the out-degree.

Irrespective of how weighting factor and network position are related to each other, groups with hierarchical and random structures always exhibit higher navigational accuracy than those groups that have no underlying social structure (figure 3*b*). Groups in which social position and weighting factor are positively correlated (i.e. the more followers an individual had the higher its weighting for its own preferred direction was) improved the most. On average hierarchical groups arrived 234 m (±386 m s.d.) closer to the goal than those groups with no network, whereas random groups improved only by 124.5 m (±546 m s.d.). When we reversed the relationship between out-degree and weighting factor, so that they were negatively correlated, we observed that groups with hierarchical social structure navigated more efficiently than those without. In that scenario, groups with random networks did not show an improvement. However, in comparison with groups in which *w* and out-degree were positively correlated, navigational performance decreased significantly when social-leaders paid less attention to their social surroundings (hier: *W* = −277654, *p* < 0.001; rand: *W* = −187216, *p* < 0.001; figure 3*b*).

### 3.3. Relationship between social structures and increased navigational error

**Figure 4.**
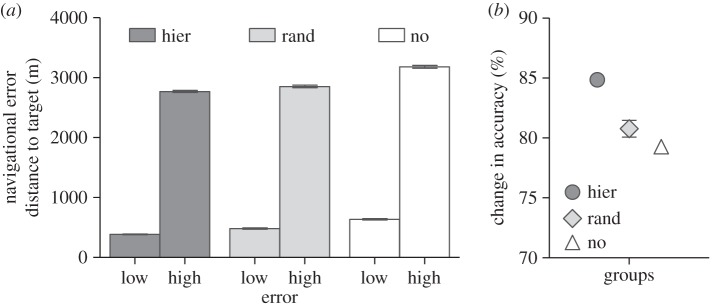
(*a*) Navigational accuracy for differently organized groups with low and high navigational error (mean ± s.e.m.). (*b*) Relative change in accuracy (mean ± s.e.m.) between low and high error groups. Groups with random, hierarchical or no networks are shown as light grey diamonds, dark grey circles or white triangles, respectively.

As a final examination of our different group structures, we increased the navigational error within the group. To explore the largest possible difference between differently structured groups, we used groups in which weighting factor and out-degree are positively correlated. In all previous simulations, the error corresponded to a random angle taken from a circular wrapped Gaussian distribution, centred on 0, with standard deviation *σ* = 0.01 radians. To further analyse the effect of less informed individuals, we increased the mean to 0.1 radians. As expected, the navigational accuracy of all groups decreased by at least 79% (figure 4*a*,*b*). Also, although the accuracy of group with hierarchical networks decreased the most (on average 84 ± 8.4% s.d.), their navigational performance was still better than that of randomly structured groups (*W* = 272 036, *p* = 0.017). Interestingly, when leading individuals also exhibited the largest navigational error (i.e. positive correlation between number of followers, *w*, and noise), we found that the advantage of groups with hierarchical structures disappeared. In this case, there was no difference in navigational performance between groups with random and hierarchical networks (*W* = 258 381, *p* = 0.37).

## 4. Discussion

We employed a mathematical modelling approach to study how various forms of underlying social organization may affect navigational performance during collective movement of goal-oriented animals. We simulated small groups of navigating individuals with varying social network structures and measured navigational accuracy within the resultant ‘flocks’. Previously, also using mathematical modelling, it has been shown that undirected networks can reduce navigational error in large groups [23]. Here, we further explored this effect on small groups that better resemble pigeon flocks from previous empirical studies [27–29].

We firstly confirmed the finding by Bode *et al.* [23] that the introduction of an underlying social structure increases navigational accuracy. Furthermore, we found that in groups with hierarchical social structures, navigational performance decreases with the average out-degree value. Groups achieved highest accuracy when each individual had only one strong connection, with more connected groups showing a decreased performance. Although individuals still reacted to conspecifics to which they had only weak connections, the group exhibited a predetermined ‘chain’ along which movement decisions cascaded. By paying attention to more than one member of the group, information about how to reach the target accurately might be weakened by predominantly distracting interactions with conspecifics. Also, with higher average degrees, the groups come to more closely resemble a homogeneous network, similar to the no network case. As a consequence, there is little improvement in navigation. Many animal species exhibit hierarchical group organization based on, for example, dominance [39]. Our findings suggest that such groups move most accurately when each member's movement decisions are mainly influenced by one other focal individual (e.g. the dominant male).

We also examined the effect of varying the weighting each individual places on its own information about the preferred target in relation to the weighting it places on social interactions, which can also be referred to as individual ‘assertiveness’ [40]. In general, as assertiveness increased, navigational accuracy increased for groups both with and without underlying social structure. However, when each member of the group was heavily influenced by its own preferred direction while attending less to the movements of conspecifics, preferred attachments became insignificant factors. As all individuals have the same preference for navigation, the only difference between the different networks is the individuals' social information. Individuals at the top of the network react equally to all group members, just like there was no social structure. As in previous studies [24], we found that leading individuals are located centrally in the group. Yet, this was true only for hierarchical networks. Central individuals can interact with all other individuals surrounding them, this allows them to navigate more efficiently because gathering directional estimates from all group members can reduce the navigational error [14]. Thus, a hierarchical structure allows more efficient ‘averaging out’ of errors. Groups with random networks do not have this property. This also holds true when the navigational error of the group is increased. Although higher navigational error within the group leads to less-efficient navigation, groups with hierarchical networks can compensate for such increase better than groups with random networks. This, however, is only true when the noise is randomly distributed across the group. When those individuals that are most influential also exhibit the highest error, hierarchical structures lose their advantage over randomly structured groups. The higher error interferes with the information of leading individuals and propagates through the group, thereby decreasing the accuracy of the entire group.

High assertiveness can be observed in animals for which reaching the target is most crucial (e.g. food-deprived animals [41] or lactating females [42]). Nonetheless, groups with unassertive members benefited more from hierarchical organization than from no or random networks. Groups in which social interactions are highly influential can gain navigational benefits from a transitive social structure. Past work has suggested that a higher weighting on the preferred direction can result in more efficient navigation. And that individuals can increase their influence on the group's movements by changing their assertiveness, or weighting [40]. Here, we found that navigation was most accurate in groups in which the individuals with the most social-followers were also highly assertive. Interestingly, hierarchical organization became less effective when highly followed individuals paid less attention to their target information (unassertive social-leaders). As described before such social-leader tends to be located at the centre of the group, but, because they disregard the navigational choices of their surroundings, they do not benefit from averaging information from the other group members. As a consequence, the mismatch between assertiveness and network position results in less-efficient navigation. King *et al.* [43] found that groups of chacma baboons (*Papio ursinus*) failed to visit food patches when the dominant male was guarding oestrus females [43]. The priority of the male changed from foraging to mate-guarding—a phenomenon which can be compared with the unassertive social-leader scenario in the present simulation.

Empirical studies have shown that individuals' movement decisions are strongly influenced by their social relationships [44,45]. In species with strong dominance hierarchies such as mountain gorillas (*Gorilla beringei beringei*) or wolves (*Canis lupus*), the alpha male consistently determines the group's movements [46,47]. Similarly, theoretical work has shown that collectively migrating groups can consist of a small group of actively navigating individuals, while the greater part of the group adopts socially facilitated movement behaviour [48]. However, the present work shows that if those individuals that have many social-followers pay less attention to their preferred directions, the group gains lower navigational benefits from underlying social structures. Hence, we can assume that if group performance is to be maximized, individual assertiveness and position within the social network should correlate with one another.

The risk of group fragmentation increases with individual assertiveness, as reaching the target becomes a higher priority than staying in a group [40]. As all individuals in our groups were informed about the target goal, we observed fragmentation only in groups with random network structures and only when the weighting factor was low (when individuals put more weight on social interactions). Randomly generated networks can allow the formation of several unconnected subgroups, which in turn may decrease the group's overall navigational performance. Such subgroups seem highly likely in animal groups, because many preferred interactions may be between pairs of individuals (e.g. sexual partners, parents–offspring). Hence, our results suggest that the performance of moving animal groups is likely to be critically affected by the group's structure.

Nagy *et al.*'s [27] study examining pigeon flock dynamics not only found a well-defined leadership hierarchy among flock members in terms of the initiation and copying of small-scale directional changes, but also that individuals' spatial positions within the flock correlated with their place in the hierarchy. It has been shown in several studies that an individual's spatial position within the group is linked to its position in the underlying social structure [24,49]. Here, we also found that highly followed individuals can be found closer to the centre of the group. This way, they can interact with more individuals, which, in turn, improves the navigation of the group. Crucially, social dominance exhibited outside of a navigational context appears uncorrelated with leadership, despite both exhibiting transitive multilevel hierarchical organization [50]. Nonetheless, the existence of the separate leadership hierarchy represents a specific form of organization that, if in agreement with the social structure, should have a positive effect on navigational performance. Along similar lines, as modelling work has shown that hierarchical knowledge distribution ensures the best group performance [26], it would be interesting to examine the extent to which competence and social structure map onto one another in a variety of contexts. However, which aspect of an individual determines the type and number of its preferential attachments may vary between species and depend on many different features. For example, it has been shown that previous histories of encounters between pairs of pigeons have a carry-over effect on their behaviour with respect to each other even in a larger group [28]. Our adjustments to this model mainly focus on the specific interaction and movement parameters, whereas the core features remain identical to the original model [12] that has been show to match empirical data for a variety of different species [29,51]. However, it would be particularly interesting to investigate model features further so as to explore the impact of social structure on navigational performance of bird species that, for example, fly in formation [52], or that are strongly affected by navigational experience [53].

In summary, our results confirm theoretical predictions that the navigational accuracy of a group will depend strongly on detailed aspects of its social organization, and furthermore suggest which of several alternatives produces the best performance in small navigating groups. Our results have broader implications for studies on collective navigation and motion because they show that only by considering a group's social system we can fully elucidate the dynamics and advantages of joint movements.
